# Factors associated with poor outcomes of continuous renal replacement therapy

**DOI:** 10.1371/journal.pone.0177759

**Published:** 2017-05-24

**Authors:** Chih-Chin Kao, Ju-Yeh Yang, Likwang Chen, Chia-Ter Chao, Yu-Sen Peng, Chih-Kang Chiang, Jenq-Wen Huang, Kuan-Yu Hung

**Affiliations:** 1 Division of Nephrology, Department of Internal Medicine, Taipei Medical University Hospital, Taipei, Taiwan; 2 Department of Internal Medicine, School of Medicine, College of Medicine, Taipei Medical University, Taipei, Taiwan; 3 Graduate Institute of Clinical Medicine, College of Medicine, Taipei Medical University, Taipei, Taiwan; 4 Division of Nephrology, Far Eastern Memorial Hospital, New Taipei City, Taiwan; 5 Department of Quality Management Center, Far Eastern Memorial Hospital, New Taipei City, Taiwan; 6 Department of Industrial Management, Oriental Institute of Technology, New Taipei City, Taiwan; 7 Institute of Population Health Sciences, National Health Research Institutes, Zhunan, Taiwan; 8 Division of Nephrology, Department of Internal Medicine, National Taiwan University Hospital, Taipei, Taiwan; University of Nottingham, UNITED KINGDOM

## Abstract

Continuous renal replacement therapy (CRRT) is one of the dialysis modalities for critically ill patients. Despite intensive dialysis care, a high mortality rate is found in these patients. Our objective was to investigate the factors associated with poor outcomes in these patients. We conducted a retrospective cohort study using the National Health Insurance Research Database. Records of critically ill patients who received CRRT between 2007 and 2011 were retrieved, and the patients were categorized into two groups: those with acute kidney injury (AKI) and those with history of end-stage renal disease (ESRD). Our primary and secondary outcomes were in-hospital mortality and long-term survival and non-renal recovery (long-term dialysis dependence), respectively, in the AKI group. We enrolled 15,453 patients, with 13,204 and 2249 in the AKI and ESRD groups, respectively. Overall, 66.5% patients died during hospitalization. In-hospital mortality did not differ significantly between groups (adjusted odds ratio, 0.93; 95% CI, 0.84–1.02). Age, chronic liver disease, and cancer history were identified as independent risk factors for in-hospital mortality in both groups. Hypertension was associated with higher risk of in-hospital mortality in patients with AKI. Age, coronary artery disease, and admission to the medical intensive care unit (MICU) were risk factors for long-term dialysis dependence in patients with AKI. Patients with AKI and ESRD have similarly poor outcomes after CRRT. Older age and presence of chronic liver disease and cancer were associated with higher mortality. Older age, presence of coronary artery disease, and admission to MICU were associated with lower renal recovery rate in patients with AKI.

## Introduction

Continuous renal replacement therapy (CRRT) is frequently used in patients admitted to intensive care units (ICUs), with 5%–6% of ICU patients developing acute kidney injury (AKI), requiring dialysis [1]. Depending on the hospital type and location[2]^2^[Ronco, Zanella et al. 2001] (2), up to 80% of patients undergo CRRT as the treatment modality [3]. The estimated median cost of CRRT is USD 300 per day, which is higher than that of intermittent renal replacement therapy [4, 5]. In addition, the mortality rate of these patients is as high as 50.6% [6]. The high medical cost and mortality rate warrant careful selection of patients for this treatment. However, current knowledge regarding which patients will most likely benefit from CRRT is limited.

The outcomes of CRRT in patients with AKI and in patients with preadmission ESRD are controversial. Allegretti et al. [3] reported a comparable mortality rate of 61% and 54% in patients with AKI and those with preadmission ESRD, respectively, whereas a meta-analysis revealed that short- and long-term outcomes are more favorable in patients with ESRD admitted to ICUs than in dialysis-requiring patients with AKI [7]. The risk factors associated with poor prognosis in patients with AKI and in patients with preadmission ESRD are different. In a prospective cohort analysis at a single medical center [3], the factors predicting higher mortality in patients with AKI were age > 60 years, higher lactate levels, and liver disease, whereas factors for poor outcomes in patients with ESRD were liver disease and admission to the medical ICU (MICU). Another study reported that the severity of comorbidities and non-renal organ dysfunction are major risk factors for in-hospital mortality [7]. CRRT in patients with AKI and in those with preadmission ESRD represent different clinical scenarios. Therefore, studying the prognosis of CRRT independent of preexisting renal disease is crucial.

Only 25% of patients with AKI who underwent CRRT survived and were dialysis-free after hospital discharge [3]. The factors responsible for dialysis dependence in patients with AKI-requiring CRRT have been evaluated in previous studies; however, factors predicting renal recovery in such patients have not been established [3]. Fortrie et al. [8] reported relatively favorable renal recovery in patients without comorbidities compared with patients with comorbidities. Understanding the likelihood of renal recovery in critically ill patients with AKI can help in choosing the appropriate supportive care measures, such as CRRT. Our database provides longitudinal follow-up data from preadmission comorbidity to medical resource consumption after hospital discharge for a large patient population. The present study was a nationwide, population-based study to determine the survival difference and risk factors associated with overall and renal outcomes in CRRT-requiring patients.

## Materials and methods

### Data source

This was a national, population-based cohort study in Taiwan. The National Health Insurance (NHI) program in Taiwan is a single-payer system covering 99% of the Taiwanese population (23 million people) [9]. The NIH administration manages and releases the NHI data as National Health Insurance Research Database (NHIRD) for research. A cohort of 2 million patients who underwent dialysis, diagnosed with chronic kidney disease, AKI, or severe neurological diseases between 1997 and 2011 was built up for a project of diseases in the kidney and brain. The study cohort was a random sample from all individuals with aforementioned conditions, with a sampling fraction of 71% [10]. The NHIRD contains all the outpatient and inpatient registration files and claims data, including demographic data, prescription data, diagnostic codes (based on the International Classification of Diseases, Ninth Revision, Clinical Modification, ICD-9-CM), and procedure codes. Because the data in the NHIRD are deidentified, informed consent was waived; this study was approved by the Institutional Review Board of Far Eastern Memorial Hospital (102167-E).

### Patient selection

Among this 2 million patient cohort, we further built up a cohort for the study. Patients aged older than 20 years and who received CRRT during the index hospitalization between January 1, 2007, and December 31, 2011, were enrolled (n = 16,418). We excluded patients who received CRRT during the subsequent hospitalization (n = 820), and those who underwent renal transplantation before the index hospitalization (n = 145). The eligible patients (n = 15,453) were classified into two groups: patients without preadmission dialysis history (AKI group) and patients who underwent outpatient hemodialysis or peritoneal dialysis within 2 months before their index hospitalization (ESRD group). All patients were followed up until death, lost to follow-up, or the end of the study (December 31, 2011).

### Data collection

Baseline demographics and comorbidities were collected. Comorbidities were identified using the ICD-9-CM codes. The severity of comorbidities was measured using the Charlson comorbidity index [11] and a comorbidity index that excluded renal disease [12]. The socioeconomic characteristics of the patients, includingeconomic status (low or high, based on the payment of insurance amount of 588 USD per month) and hospital type (medical center or non-medical center), were analyzed. The hospital type consisted of three categories, including local clinics, and regional hospital and medical center, based on an assessment system by the State Joint Commission on Hospital Accreditation. The department of admission was categorized as MICU, surgical ICU (SICU), and others.

The primary and secondary outcomes were in-hospital mortality, and long-term survival of all patients and renal recovery in AKI group patients, respectively. For the outcome of renal recovery, we defined patients who undergo dialysis therapy 60 to 120 days after discharge as long-term dialysis dependence. Because death acts as a completing risk for dialysis dependence, we determined a composite outcome of in-hospital mortality: death within 60 days of discharge or long-term dialysis dependence.

### Statistical analysis

The collected data were analyzed by performing *t* tests and chi-square tests for continuous and categorical variables, respectively. The logistic regression model was used to determine the risk factors associated with in-hospital mortality in all patients, AKI group, and ESRD group. The interaction between preadmission ESRD status and risk factors for in-hospital mortality was tested. Multivariable Cox-regression analysis was performed for long-term survival. Logistic regression analysis was performed to determine the risks of non-renal recovery (long-term dialysis dependence). Adjusted variables included age, sex, comorbidity index, economic status, hospital type, ICU, and all comorbidities. All statistical analyses were performed using SAS 9.3 software (SAS Institute Inc, Cary, NC, USA). P < 0.05 was considered statistically significant.

## Results

### General demographics

**Fig 1:**
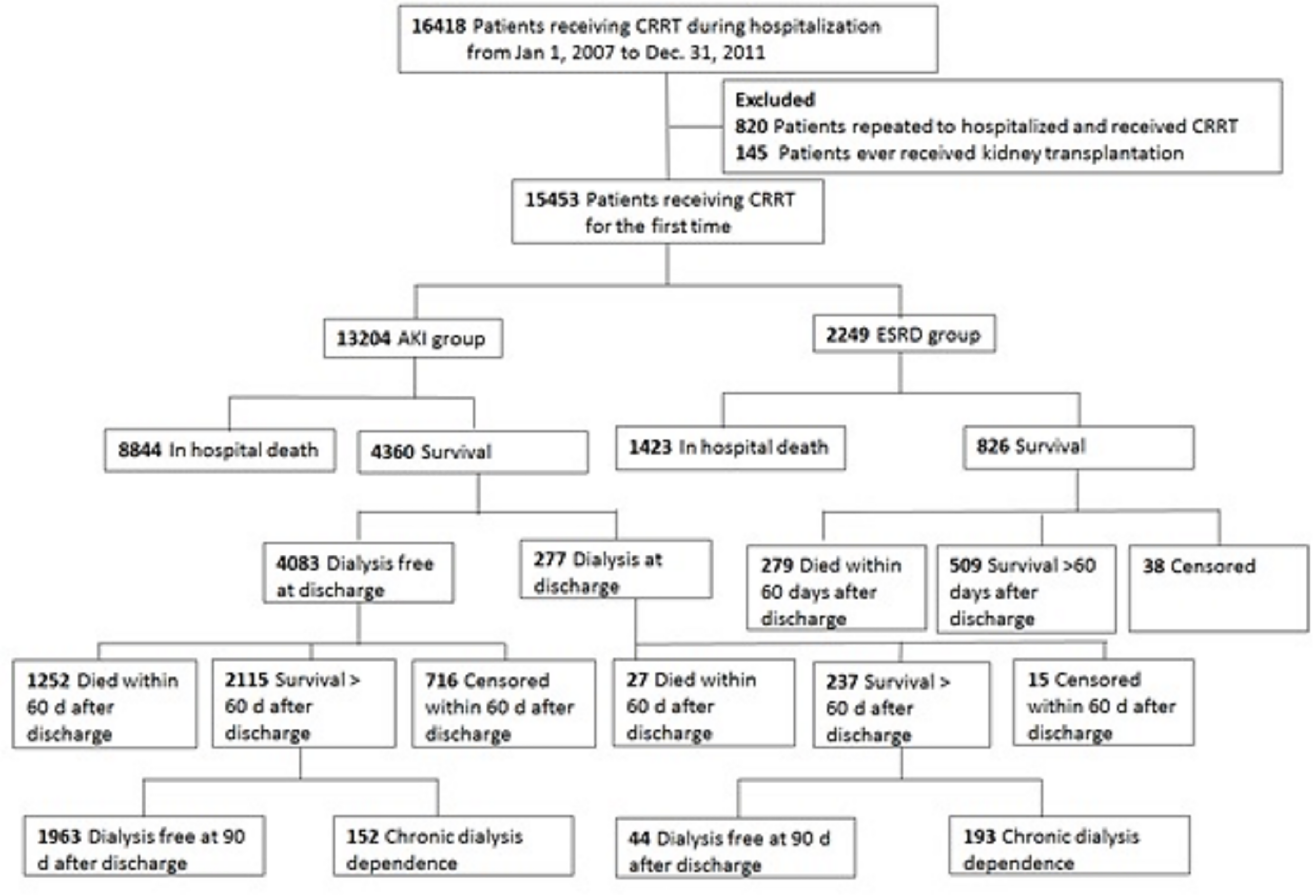
Flowchart of patient selection. AKI: acute kidney injury, CRRT: continuous renal replacement therapy, ESRD: end-stage renal disease.

The study enrolled 13,204 and 2249 patients in the AKI and preadmission ESRD groups, respectively. **Fig 1** illustrates the patient selection process. Several baseline demographic characteristics differed between the two groups **(Table 1)**. The mean age was 66.8 and 68.0 years in the AKI and ESRD groups, respectively. The majority of patients in the AKI group were male, and patients in the ESRD group had a similar comorbidity index [12]. More patients who received CRRT at the SICU were in the AKI group (28.1%) than in the ESRD group (24.2%).

**Table 1:** Baseline characteristics of patients undergoing continuous renal replacement therapy (CRRT) in the AKI and ESRD groups.

Characteristics	AKI (n = 13,204)	ESRD (n = 2249)	P
Mean age in years (std)	66.8 (15.9)	68.0 (12.2)	<0.01
Male, n (%)	8,571 (64.9)	1,118 (49.7)	<0.01
Comorbidity index, mean (std)			
Charlson	2.9 (2.6)	4.0 (2.5)	<0.01
Liu (12)	2.6 (2.4)	2.6 (2.2)	0.57
Economic status			
Low	6758 (51.4)	1187 (52.8)	0.22
Institute level			
Medical center	9538 (72.4)	1576 (70.1)	0.04
ICU			
MICU	8605 (65.2)	1490 (66.3)	<0.01
SICU	3710 (28.1)	545 (24.2)	
Others	889 (6.7)	214 (9.5)	
Comorbidity			
Hypertension	7066 (53.5)	1275 (56.7)	0.03
Diabetes	5178 (39.2)	1256 (55.9)	<0.01
Coronary artery disease	4216 (31.9)	1061 (47.2)	<0.01
Cerebral vascular disease	1951 (14.8)	392 (17.4)	<0.01
Heart failure	3342 (25.3)	814 (36.2)	<0.01
Arrhythmia	2221 (16.8)	442 (19.7)	<0.01
Valvular heart disease	1376 (10.4)	244 (10.9)	0.54
Peripheral vascular disease	1082 (8.2)	365 (16.2)	<0.01
COPD	2325 (17.6)	325 (14.5)	<0.01
Chronic liver disease	3242 (24.6)	435 (19.3)	<0.01
Cancer	2883 (21.8)	357 (15.9)	<0.01

Abbreviations: std: standard deviation, AKI: acute kidney injury, COPD: chronic obstructive pulmonary disease, ESRD: end-stage renal disease, ICU: intensive care unit, MICU: medical intensive care unit, SICU: surgical intensive care unit.

### In-hospital mortality

The in-hospital mortality rate in the AKI group (67.0%) was higher than that in the ESRD group (63.3%), but this finding was not significant after adjusting the baseline factors (adjusted OR 0.93, 0.84–1.02, P = 0.15). Multivariable logistic regression showed that the risks of in-hospital mortality included age, MICU admission, peripheral vascular disease, chronic obstructive pulmonary disease (COPD), chronic liver disease, and cancer in all patients (**Table 2**). Diabetes mellitus (DM), congestive heart failure (CHF), and admission to medical center were associated with relatively favorable outcomes.

**Table 2:** Multivariate model for determining risk factors for in-hospital mortality in all patients, ESRD group, and AKI group.

Factor	Crude OR			Inter.	Adjusted OR			Inter.
	All	ESRD	AKI	P	All	ESRD	AKI	P
ESRD vs. AKI	0.85 (0.77–0.93)	N/A	N/A	N/A	0.93 (0.84–1.02)	N/A	N/A	N/A
Age	1.01 (1.00–1.01)	1.01 (1.01–1.02)	1.01 (1.00–1.01)	0.06	1.01 (1.01–1.01)	1.02 (1.01–1.02)	1.01 (1.01–1.01)	0.06
Male vs. female	1.09 (1.02–1.17)	0.90 (0.76–1.07)	1.11 (1.03–1.20)	0.08	1.04 (0.97–1.12)	0.94 (0.79–1.12)	1.06 (0.98–1.15)	0.08
Low vs. high economic	0.9 (0.92–1.06)	1.14 (0.96–1.35)	0.96 (0.90–1.04)	0.04	1.01 (0.94–1.08)	1.18 (0.99–1.40)	0.98 (0.91–1.06)	0.04
Medical center	0.80 (0.74–0.86)	0.80 (0.66–0.97)	0.80 (0.74–0.87)	0.74	0.80 (0.74–0.86)	0.82 (0.68–1.00)	0.79 (0.73–0.86)	0.74
MICU	1.23 (1.15–1.32)	1.21 (1.01–1.45)	1.24 (1.15–1.34)	0.93	1.18 (1.10–1.27)	1.15 (0.5–1.38)	1.18 (1.09–1.28)	0.93
HTN	1.04 (0.97–1.11)	0.84 (0.71–1.00)	1.08 (1.00–1.16)	0.03	1.07 (0.99–1.15)	0.89 (0.74–1.06)	1.12 (1.03–1.22)	0.03
DM	0.83 (0.77–0.88)	0.80 (0.67–0.95)	0.84 (0.78–0.91)	0.64	0.81 (0.75–0.87)	0.84 (0.70–1.01)	0.79 (0.73–0.86)	0.64
CAD	0.8 (0.83–0.95)	0.81 (0.68–0.96)	0.92 (0.85–0.99)	0.35	0.97 (0.89–1.05)	0.87 (0.72–1.05)	0.99 (0.91–1.08)	0.35
CVD	0.93 (0.84–1.01)	0.80 (0.64–1.00)	0.96 (0.87–1.06)	0.26	0.95 (0.87–1.05)	0.86 (0.68–1.08)	0.98 (0.88–1.09)	0.26
CHF	0.87 (0.81–0.94)	0.91 (0.76–1.08)	0.88 (0.81–0.95)	0.70	0.92 (0.85–1.00)	0.97 (0.80–1.18)	0.91 (0.83–1.00)	0.70
PVD	1.01 (0.90–1.13)	1.14 (0.90–1.44)	0.99 (0.87–1.13)	0.27	1.14 (1.01–1.28)	1.23 (0.97–1.57)	1.09 (0.95–1.25)	0.27
COPD	1.31 (1.19–1.43)	1.05 (0.82–1.35)	1.34 (1.22–1.48)	0.11	1.22 (1.11–1.35)	1.00 (0.78–1.2)	1.27 (1.14–1.41)	0.11
Liver	1.71 (1.57–1.86)	1.67 (1.33–2.11)	1.70 (1.56–1.86)	0.53	1.69 (1.55–1.84)	1.67 (1.32–2.12)	1.69 (1.54–1.86)	0.53
Cancer	1.90 (1.74–2.08)	1.50 (1.17–1.92)	1.96 (1.78–2.15)	0.03	1.75 (1.60–1.92)	1.33 (1.03–1.71)	1.82 (1.65–2.01)	0.03

Abbreviations: OR: odds ratio, inter.: interaction, DM: diabetes mellitus, CAD: coronary artery disease, CHF: congestive heart failure, COPD: chronic obstructive pulmonary disease, CVD: cerebrovascular disease, HTN: hypertension, MICU: medical intensive care unit, PVD: peripheral vascular disease.

The risk factors for in-hospital mortality in the AKI group were almost similar to that in all patients. The risk profiles of in-hospital mortality for the ESRD group included age, chronic liver disease, and cancer history. Formal interaction tests indicated that the history of hypertension and cancer was associated with a relative higher risk of in-hospital mortality in patients with AKI than in patients with ESRD.

### Long-term survival

**Fig 2:**
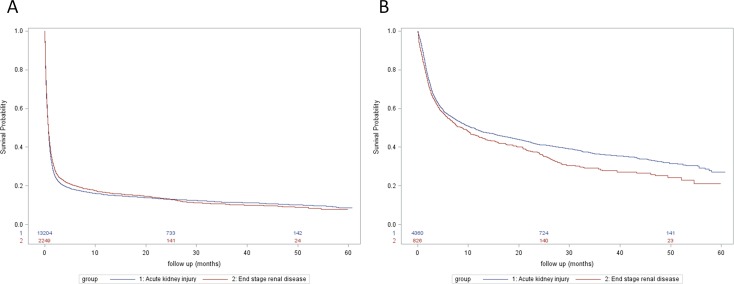
Kaplan–Meier survival curves for long-term survival. (A) Total population (HR 0.98, 95% CI 0.93–1.03; adjusted HR 1.02, 95% CI 0.97–1.07) (B) Patients who survived and discharged from index hospitalization (HR 1.16, 95% CI 1.05–1.28; adjusted HR 1.21, 95% CI 1.09–1.34).

Long-term survival was not significantly different after adjustment (adjusted HR 1.02, 95% CI 0.97–1.07) (**Fig 2A**). Analyzing the patients who survived from the index hospitalization, the long-term survival in the AKI group was higher than that in the ESRD group (adjusted HR for death of patients with preadmission ESRD compared with patients with AKI: 1.21, 95% CI 1.09–1.34). Approximately one-third of patients in each group died (30.6% in AKI group, 33.7% in ESRD group) within 60 days of discharge. (**Fig 2B**).

### Long-term dialysis dependence

Of the 4360 patients with AKI who survived, 277 (6.4%) remained dependent on dialysis at the time of discharge. At 60 days post-discharge, 345 (14.7%) of the 2352 patients who survived were dependent on dialysis (non-renal recovery, long-term dialysis dependence). Multivariate logistic analysis revealed that age, MICU admission, and coronary artery disease were associated with long-term dialysis dependence (**Table 3**); COPD and liver disease were associated with lower risk of dialysis dependence. A composite outcome analysis showed that COPD and liver disease were no longer a protective factor for long-term dialysis dependence.**(Table 3)**

**Table 3:** Risk factors associated with long-term dialysis dependence and composite outcomes* in the AKI group.

Factor	Long-term dialysis dependence		Composite outcome	
	Crude OR	Adjusted OR	Crude OR	Adjusted OR
Age	1.02 (1.01–1.03)	1.02 (1.01–1.02)	1.01 (1.01–1.02)	1.02 (1.01–1.02)
Male vs. female	0.74 (0.59–0.93)	0.86 (0.68–1.10)	1.07 (0.97–1.18)	1.04 (0.94–1.16)
Low vs. high socioeconomic level	0.97 (0.77–1.22)	0.89 (0.70–1.12)	0.94 (0.86–1.04)	0.95 (0.86–1.04)
Center	0.85 (0.66–1.10)	0.87 (0.67–1.13)	0.83 (0.74–0.92)	0.83 (0.74–0.93)
MICU	1.78 (1.39–2.28)	1.73 (1.34–2.25)	1.46 (1.33–1.61)	1.37 (1.24–1.51)
HTN	1.21 (0.96–1.52)	0.90 (0.69–1.17)	1.01 (0.92–1.11)	0.94 (0.84–1.05)
DM	1.18 (0.93–1.48)	0.96 (0.75–1.24)	0.78 (0.71–0.86)	0.71 (0.64–0.79)
CAD	1.86 (1.47–2.34)	1.71 (1.31–2.21)	0.97 (0.87–1.07)	1.01 (0.90–1.14)
CVD	0.96 (0.70–1.33)	0.85 (0.61–1.18)	0.93 (0.82–1.06)	0.94 (0.82–1.08)
CHF	1.57 (1.23–2.01)	1.24 (0.95–1.63)	1.00 (0.90–1.12)	1.03 (0.91–1.16)
PVD	1.15 (0.78–1.70)	1.22 (0.81–1.83)	0.97 (0.81–1.15)	1.10 (0.92–1.31)
COPD	0.86 (0.60–1.23)	0.67 (0.46–0.97)	1.53 (1.33–1.75)	1.30 (1.12–1.50)
Liver	0.51 (0.35–0.75)	0.59 (0.40–0.88)	1.76 (1.55–1.99)	1.82 (1.59–2.07)
Cancer	0.76 (0.51–1.14)	0.82 (0.55–1.24)	2.49 (2.15–2.88)	2.28 (1.97–2.65)

*Composite outcomes: Combined outcome of in-hospital mortality, death within 60 days after discharge and long-term dialysis dependence. Abbreviations: DM: diabetes mellitus, CAD: coronary artery disease, CHF: congestive heart failure, COPD: chronic obstructive pulmonary disease, CVD: cerebrovascular disease, HTN: hypertension, MICU: medical intensive care unit, PVD: peripheral vascular disease.

## Discussion

Our study yielded a comparable in-hospital mortality rate and long-term survival rate between AKI and ESRD groups, which is consistent with the results obtained by Allegretti et al. [3]. Dialysis-requiring AKI may reflect a systemic insult and it is often associated with multi-organ dysfunction [1, 13], which might increase the risk of mortality. The findings suggest that clinicians should continue with CRRT in appropriately selected patients with ESRD, if clinically indicated, because the risk of mortality is not higher than in CRRT-requiring patients with AKI. Arulkumaran et al. [7] reported that patients with ESRD admitted to ICU had better outcomes than AKI-dialysis patients. The mean ICU mortality rate was 21.4%, which was much lower than that of our cohort. In addition, all of our patients underwent CRRT, which is different from that in the systemic review, mainly by intermittent hemodialysis [7]. Patients undergoing CRRT were mostly critically ill and hemodynamically unstable, which was evidenced by the high mortality rate [3, 14]. To summarize, different disease severity and dialysis modality (CRRT or conventional dialysis) may be an important regulator of patients’ survival between critically ill ESRD and AKI-dialysis patients.

In this study, the long-term survival after index hospitalization was higher in patients with AKI than in patients with preadmission ESRD. Although in-hospital mortality is high in AKI-dialysis patients, the patients who survived at discharge have high probability of renal recovery and subsequently better long-term outcome compared with patients with ESRD. More than 85% of AKI-requiring dialysis patients who survived at discharge have renal function recovery [15]. Another study performed in a tertiary referral center reported lower mortality rate in AKI CRRT patients (28.4%, 81/285) than in ESRD CRRT patients (39.7%, 25/63) during the follow-up period after discharge [3]. In our study, among 2352 patients with AKI who survived >60 days after discharge, only 345 (14.7%) patients were still dialysis dependent, which was highly comparable to the above study by Duran. A high proportion of renal recovery in AKI subgroup may account for the better long-term survival in AKI-surviving patients.

In this study, 65% of patients with AKI were male. Uchino S et al [1] showed a total of 64% patients male (1105/1738) in a multi-national, multi-center study. Ours is quite similar to this study. Another French prospective, multi-center cohort also showed similar findings with more than 66% of severe patients with AKI male [16]. To summary, from our study and literature reviews, the majority of patients who developed severe AKI were male. Age, liver disease, and MICU are established risk factors for in-hospital mortality in both AKI and patients with ESRD receiving CRRT [6], [17]. In cirrhotic patients, the risk of in-hospital mortality markedly increases as AKI worsens [18]. In addition, hepatorenal syndrome is a major contributing factor for AKI in patients with cirrhosis, resulting in poor outcome [19, 20]. Chronic liver disease also presents a risk of mortality in CRRT-requiring patients with ESRD [3]. Sepsis and septic shock are the most frequent cause of AKI in critically ill patients admitted to the MICU; these infections increase the mortality rate [21]; by contrast, patients with AKI admitted to the SICU were often caused by cardiothoracic and vascular surgery and associated with lower mortality (~5%) [22]. Thus, the etiology of AKI in patients admitted to the MICU increases the risks of in-hospital mortality.

An underlying history of cancer has been associated with increased mortality rate in patients with AKI [23] and ESRD admitted to the ICUs [24]. The poor outcomes of patients with cancer may arise from their impaired immunity and nutritional status [25]. The negative effect of cancer on in-hospital mortality in the AKI group was significantly stronger than that in the ESRD group (adjusted OR 1.82 vs. 1.33, P for interaction, 0.03). The prevalence of cancer in patients with AKI was much higher than that in patients with ESRD undergoing CRRT (21.8% vs. 15.9%), thus we proposed that patients with ESRD and advanced cancer may more likely choose palliative care over intensive care with CRRT at the ICU, and the proportion of patients with advanced cancer might be even more lower in the ESRD group, which may explain the relative higher risk of mortality in the AKI group. However, cancer stage-stratified analysis in the two groups was limited by the dataset.

Hypertension was identified as a risk factor of mortality in the AKI group, but not in the ESRD group (Table 2). Hypertension has not been identified as a risk factor for critically-ill patients undergoing CRRT in previous studies [3, 26]. Additional complications might force hypertensive patients with AKI to undergo CRRT. Patients with AKI with underlying hypertension might be prone to organ system failures, such as cardiovascular [27], neurological [28], pulmonary dysfunction [29], and higher mortality. Further research is necessary to elucidate whether a reverse epidemiology of hypertension and in-hospital mortality was observed in patients with ESRD undergoing CRRT.

Admission to a medical center, DM, and CHF were associated with lower in-hospital mortality. A cohort study using the Japan National Database reported that academic hospitals carry lower risks of in-hospital mortality in patients with AKI who require CRRT [6]. Hospital volume has been shown to influence the outcomes in patients undergoing surgery and in critically ill patients [30, 31]. Medical staff familiar with the CRRT procedures, such as the initial set-up and circuit problem resolution, can benefit the patients by helping them more easily achieve the target CRRT dose. A recent study reported that strong expertise of the CRRT team is associated with favorable clinical outcomes because of the higher quality of care [32]. Although DM and CHF have been reported to be associated with poor outcome in dialysis patients [33], some studies [3] have reported that DM and CHF are not risk factors for mortality in patients receiving CRRT. Diabetic patients might have a low threshold for initiating CRRT as they often have difficulties in body fluid management and therefore easily develop pulmonary edema during AKI. Similarly, the association between CHF and lower in-hospital mortality could be due to this indication bias, which implied that early initiation of CRRT may be protective in this patient group.

The dialysis-dependence rate of patients with AKI at discharge and at 60 days post-discharge was 6.4% (277/4360) and 14.7% (345/2352), respectively. A previous study showed that dialysis dependence in dialysis-requiring patients with AKI after 1 and 3 years of discharge was 19.1% and 30%, respectively [34]. A study by the Acute Renal Failure Trials Network [35] that enrolled 1124 patients with severe AKI reported that 25% of the surviving patients at 60 days post-discharge were dialysis dependent, whereas an Australian study reported that only 5.4% of the survivors were dialysis dependent at 90 days [36]. Because most patients with severe AKI died, the estimated rate of long-term dialysis dependence differed significantly from the mortality rate of the cohort.

Age, MICU admission, and coronary artery disease (CAD) were independently associated with dialysis dependence. Age has been recognized as a risk factor for poor recovery of renal function after AKI [37, 38]. The causes of AKI in MICU patients might explain the higher dialysis dependence rate than in SICU patients. For patients with CAD, the “dry lung” strategy may help patients overcome cardiac events but may cause kidney damage. Therefore, patients with AKI with cardiovascular comorbidities are less likely to have renal recovery. Chronic lung and liver diseases appear to be associated with lower long-term dialysis dependence. However, when in-hospital mortality, death within 60 days after discharge and long-term dialysis dependence were considered as composite outcomes, the favorable effect of chronic lung and liver disease on the dialysis outcome disappeared.

The strength of our study is a nationwide database that is representative of more than 99% of the residents of Taiwan. The study involved a comprehensive survey and longitudinal follow-up of several characteristics from preadmission characteristics to status after discharge. However, the use of the NHIRD dataset entailed several limitations: (i) inability to identify indications for CRRT, cause of AKI, and severity of comorbidities; (ii) lack of information on CRRT prescription and delivery, such as time lapse between onset of AKI and initiation of dialysis, dialysis intensity, vascular access, and ultrafiltration volume; and (iii) unavailability of laboratory results. Inadequate information on these crucial parameters restricted us from building an effective prediction model. An observational study could only predict the association between factors and outcomes but does not determine the causal relationship.

In conclusion, no significant difference of in-hospital and long-term mortality rate was seen in patients with AKI and ESRD. Old age, liver disease, and MICU were risk factors for in-hospital mortality in patients with AKI and ESRD undergoing CRRT. Cancer and hypertension predicted higher mortality particularly among patients with AKI. Elderly, MICU admission, and cardiovascular comorbidity were associated with lower renal recovery rate. These findings are useful in the clinical setting and require additional studies to validate the results.
